# Comparative Effectiveness of Dipeptidyl Peptidase-4 (DPP-4) Inhibitors and Human Glucagon-Like Peptide-1 (GLP-1) Analogue as Add-On Therapies to Sulphonylurea among Diabetes Patients in the Asia-Pacific Region: A Systematic Review

**DOI:** 10.1371/journal.pone.0090963

**Published:** 2014-03-10

**Authors:** Martin C. S. Wong, Harry H. X. Wang, Mandy W. M. Kwan, Daisy D. X. Zhang, Kirin Q. L. Liu, Sky W. M. Chan, Carmen K. M. Fan, Brian C. Y. Fong, Shannon T. S. Li, Sian M. Griffiths

**Affiliations:** 1 JC School of Public Health and Primary Care, Faculty of Medicine, Chinese University of Hong Kong, Hong Kong SAR, China; 2 General Practice and Primary Care, Institute of Health and Wellbeing, University of Glasgow, Glasgow, United Kingdom; National Institute of Agronomic Research, France

## Abstract

The prevalence of diabetes mellitus is rising globally, and it induces a substantial public health burden to the healthcare systems. Its optimal control is one of the most significant challenges faced by physicians and policy-makers. Whereas some of the established oral hypoglycaemic drug classes like biguanide, sulphonylureas, thiazolidinediones have been extensively used, the newer agents like dipeptidyl peptidase-4 (DPP-4) inhibitors and the human glucagon-like peptide-1 (GLP-1) analogues have recently emerged as suitable options due to their similar efficacy and favorable side effect profiles. These agents are widely recognized alternatives to the traditional oral hypoglycaemic agents or insulin, especially in conditions where they are contraindicated or unacceptable to patients. Many studies which evaluated their clinical effects, either alone or as add-on agents, were conducted in Western countries. There exist few reviews on their effectiveness in the Asia-Pacific region. The purpose of this systematic review is to address the comparative effectiveness of these new classes of medications as add-on therapies to sulphonylurea drugs among diabetic patients in the Asia-Pacific countries. We conducted a thorough literature search of the MEDLINE and EMBASE from the inception of these databases to August 2013, supplemented by an additional manual search using reference lists from research studies, meta-analyses and review articles as retrieved by the electronic databases. A total of nine randomized controlled trials were identified and described in this article. It was found that DPP-4 inhibitors and GLP-1 analogues were in general effective as add-on therapies to existing sulphonylurea therapies, achieving HbA1c reductions by a magnitude of 0.59–0.90% and 0.77–1.62%, respectively. Few adverse events including hypoglycaemic attacks were reported. Therefore, these two new drug classes represent novel therapies with great potential to be major therapeutic options. Future larger-scale research should be conducted among other Asia-Pacific region to evaluate their efficacy in other ethnic groups.

## Introduction

### Epidemiology & burden of diabetes mellitus

Diabetes mellitus is the most common non-communicable disease and a worldwide health crisis [1]. The previously reported prevalence of diabetes was estimated as 9.3% from the National Health and Nutrition Examination Survey (NHANES) in the United States [2]. Similar high rates were also reported in European countries such as Spain (10.3%) [3] and Poland (15%) [4]. In Asia-Pacific region, a previous study described a progressively rising trend of diabetes across the years from 2001 to 2008 in Hong Kong and the prevalence of diabetes escalated with age [5]. Another study reported that the prevalence of diabetes has reached epidemic proportions in the general population aged 20 years and older in mainland China, where 9.7% of the adult population suffered from diabetes [6]. A recent report from the International Diabetes Federation further revealed that the estimated global prevalence of diabetic patients was projected to increase from 2.8% in 2000–2007 to 9.9% in 2025–2030, affecting a total of 552 million population [7]. Such epidemiological evidence indicates that significant surge in the incidence of diabetes is expected in the next few decades. There has also been sound evidence suggesting that the rapidly escalating prevalence of diabetes has imposed a substantial economic burden at the society level, including the healthcare systems [8]. In 2011, the estimated global health care expenditure due to diabetes was US$465 million, accounting for approximately 11% of total health expenditure among adults in the world [9]. In addition, diabetes is associated with excess morbidity and mortality [10], [11] which could lead to reduction of health-related quality of life and life expectancy [12]. Its associated comorbidities increase hospitalisation rates due to the risk of long-term complications, such as coronary artery disease, stroke, diabetic neuropathy, renal failure, blindness, and peripheral vascular disease which might require limb amputation [13], [14]. Type 2 diabetes accounts for around 90% of diabetes cases and it is the most common form that is associated with ageing population, environmental changes, and health behaviour patterns. The optimal control of type 2 diabetes has therefore significantly emerged as one of the most challenging issues faced by physicians and policy-makers worldwide.

### Traditional drugs used for diabetes mellitus

Metformin has been used alone as the first line OHA for type 2 diabetes [15]–[18]. It reduces hepatic glucose output and promotes peripheral glucose uptake [19]. Its ability to reduce glycated haemoglobin (HbA1c) is dose-related [20]. However, adverse events (AEs) such as gastrointestinal disturbance and lactic acidosis were reported [17]. Sulphonylureas (SUs) and thiazolidinediones are commonly used as alternatives when metformin therapy fails [16]. SUs consist of a group of insulin secretagogues, including glibenclamide, gliclazide and glipizide, which act on K^+^-channels on beta cell plasma membrane to enhance insulin secretion [15], [21]–[24]. As SUs require functioning beta cells to work, their efficacies may only limit to early stages of type 2 diabetes. Poor durability and higher secondary failure were reported in previous studies [25]–[27]. Common adverse effects documented include significant weight gain and hypoglycaemia [28], [29]. Thiazolidinediones, on the other hand, reduce insulin resistance and increase peripheral glucose uptake through binding to the peroxisome proliferator-activated receptor-γ (PPAR γ). They also promote differentiation of preadipocytes to adipocytes to increase lipogenesis [30]. Their durability of glycaemic control is better when compared with SUs or metformin [31]. AEs including peripheral oedema caused by fluid retention [32], higher risk of having congestive heart failure [33] and weight gain markedly limit their use. Hence they are usually considered only when patients have significant risks for hypoglycaemia, or are intolerant to the medications, or when SUs are contraindicated.

Combination therapy with either SUs or thiazolidinediones and metformin is a common next step if the glycaemic target is not attained by monotherapy. This results in summation of the therapeutic effects, offering patients with extra benefits [15]–[17]. Besides, acarbose is sometimes co-administered with metformin for obese type 2 diabetic patients. It is an inhibitor of intestinal-glucosidase located at the intestinal brush border, which helps reducing the post-prandial blood glucose surge by delaying the carbohydrate absorption. However, this may lead to diarrhoea, abdominal pain and bloating.

When combination drug therapy fails to achieve therapeutic targets, insulin therapy will be initiated to provide sufficient amount of insulin for maintaining homeostasis of blood glucose [15], [34], [35]. Hypoglycaemia is a common and major adverse effect of insulin [28], which may cause brain damage in severe case.

Despite the development of the above therapies for managing diabetes, large proportion of diabetic patients did not achieve their glycaemic targets. In a European cohort of 2,023 type 2 diabetic patients who were on metformin and either SUs or thiazolidinediones, only 25.5% of the patients had adequate glycaemic control. The average HbA1c level after a mean of 2.6 years of combination oral antihyperglycaemic agent therapy was 7.2% [36]. High rates of inadequate glycaemic control were also observed in China. A cross sectional multicentre study conducted among 455 type 2 diabetic patients in China reported 45.5% of the cohort had inadequate glycaemic control with HbA1c≥6.5%. The most common combination therapy used was SUs/glinides with metformin (25.9%) [37]. Another study also demonstrated that 75% of 493 diabetic inpatients had inadequate glucose control [38]. Given the maintenance of good glycaemic control is the key to prevent diabetic complications, development of safe and effective approach to manage diabetes is of high priority. Two novel therapeutic agents, namely incretin mimetics and dipeptidyl peptidase-4 (DPP-4) inhibitors, have been developed to meet this ongoing need.

### Newer medications for diabetes –Incretin mimetics and DPP-4 inhibitors

#### Incretin mimetics

Glucagon-like peptide-1 (GLP-1) analogues or agonists are a group of drugs which mimics the effect of the endogenous hormone GLP-1. They regulate blood glucose level by stimulating insulin secretion, suppressing glucagon secretion [39], delaying gastric emptying and promoting satiety [40]. Unlike some of the OHAs that lead to weight gain and hypoglycaemia, GLP-1 analogues have been shown to produce weight loss in type 2 diabetes, and have a lower risk of causing hypoglycaemia [39]–[41]. Medications belonging to this drug class include exenatide and liraglutide.

#### Dipeptidyl peptidase 4-inhibitors (DPP4-I)

DPP4-I are a class of OHAs that regulates blood glucose level by inhibiting the action of didpeptidyl peptidase 4 (DPP-4). Under normal physiological condition, incretin hormones GLP-1 and gastric inhibitory polypeptide (GIP) are released from the endocrine cells in the small intestine upon food consumption. They stimulate glucose-dependent release of insulin [42], [43]. However, these incretin hormones are rapidly degraded by the enzyme DPP-4 [44]. This new drug class helps prolonging the incretin effects by inhibiting the action of DPP-4, resulting in enhancement in insulin secretion, suppression of glucagon secretion and improvement in beta-cell function [45].

DPP4-I are overall very well tolerated and have few side effects [45]. A potential side effect of DPP4-I is that they may slightly increase the risk of infection [46]. Also, DPP4-I are slightly less effective than incretin mimetics on the reduction of HbA1c level in some patients [45]. They are weight neutral with no effect on gastric emptying [45]. Nevertheless, DPP4-I have several advantages over incretin mimetics, including oral administration, and having a lower risk of gastrointestinal discomfort and hypoglycaemia [45]. DPP4-I can be administered with or without food, while drug interactions are rare [45]. Sitagliptin, vildagliptin, saxagliptin, linagliptin and alogliptin are medications belonging to this drug class.

### Novel therapeutic agents and their potential risks

In March 2013, the Food and Drug Administration (FDA) announced that the type 2 diabetic patients treated with the drug classes of incretin mimetics and DPP-4 inhibitors may have a higher risk of pancreatitis and pre-cancerous cellular changes [47]. These findings were supported by a small number of examination results from the patients who died from unspecified causes [47]. Therefore, there is still no conclusion about their safety risks and further investigation on the potential pancreatic toxicity associated with these two new antidiabetic agents is needed.

Whilst there is no consistent and concrete evidence against the safety profile of these two new agents, one of the important topics surrounding these novel agents includes their efficacy in achieving optimal glycaemic control among diabetic patients on OHAs, in particular SUs which are used more commonly among patients in the Asia-Pacific region. We therefore searched the existing databases to review the effectiveness of the two drug classes as add-on therapies as compared with SUs or placebo.

## Methods

### Data sources and searches

A comprehensive search was performed using a combination of medical subject headings (MeSH) and keywords in international databases including MEDLINE and EMBASE in August 2013. Original research studies were searched in the databases from their inception to August 2013 that reported the effectiveness of DPP-4 inhibitors and GLP-1 analogues as add-on therapies among diabetic patients. An additional manual search was conducted using reference lists from research studies, meta-analyses and review articles to identify other potential eligible studies. **Table 1** outlined the steps of the search process.

**Table 1:** Search Strategy.

**1**	(type 2 or type2 or type II or non-insulin dependent or adult onset).tw. (400869)
**2**	(diabet 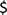 or antidiabet 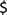 ).tw. (817946)
**3**	1 and 2 (199667)
**4**	Diabetes Mellitus, Type 2/(204183)
**5**	3 or 4 (262903)
**6**	(dpp-4 or dpp 4 or dpp4 or dpp-IV or dpp IV or dppIV or dipeptidyl-peptidase 4 or dipeptidyl peptidase 4).tw. (7463)
**7**	(incretin 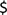 or glucose-dependent insulinotropic peptide or gastric inhibitory peptide or GIP or glucagon-like peptide-1 or GLP-1).tw. (20226)
**8**	(sitagliptin 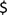 or vildagliptin 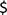 or gliptin 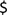 or alogliptin 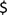 or gemigliptin 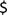 or linagliptin 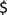 or saxagliptin 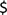 ).tw. (4061)
**9**	6 or 7 or 8 (25825)
**10**	5 and 9 (11338)
**11**	(asia 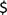 or pacific or American Samoa or Australia 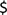 or Brunei 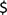 or Cambodia 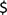 or Chin 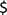 or Hong Kong or Indonesia 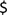 or Japan 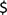 or Laos or Macau or Malaysia 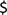 or Mongolia 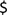 or Myanmar or New Zealand 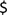 or North Korea 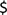 or Papua New Guinea or Philippine 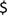 or Singapore 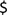 or South Korea 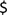 or Taiwan 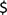 or Thailand 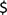 or Timor-Leste or Vietnam 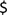 ).tw. (1238790)
**12**	10 and 11 (522)

### Study selection and quality assessment

We included studies which were designed as randomised controlled trials conducted in the Asia-Pacific region, including (1). participants having type 2 diabetes mellitus with inadequate glycaemic control at enrolment; (2). adults aged ≥18 years and were previously treated with SUs as background diabetic regimen; and that (3). the interventions aimed to assess the drug efficacy of either DPP-4 inhibitors or GLP-1 analogues as add-on anti-diabetic therapy. Only studies which were published as complete full papers in English were included. When the same patient population was reported in two or more journal articles, only the most recent and complete study was included.

All potentially relevant publications retrieved by electronic searching were reviewed independently by two investigators (HHW and MWK) based on examination of study titles and abstracts. Those studies which did not meet the inclusion criteria were excluded. The full-text of the article was obtained if either reviewer believed that the study might be eligible. Any disagreement was resolved by consensus after discussion or, when necessary, by appeal to the principal investigator (MCW).

### Data extraction and end points

A research nurse (BCF) and a research assistant (STL) independently extracted relevant information on study participants, design of interventions and controls, study duration and outcome measures from each included study by a standardised extraction form. For this systematic review, the primary outcomes were clinical values including HbA1c and fasting plasma glucose (FPG). The secondary outcomes were drug safety tolerability including the incidence and intensity of AEs, withdrawal due to accident emergency, symptomatic hypoglycaemic events, homeostasis model assessment for insulin resistance (HOMA-R), HOMA-beta cell function, etc. The details of all the included studies were shown in **Table 2**.

**Table 2:** Studies included in the present review.

Studies	Patients	Intervention	Control	Major Outcomes	Ethnicity	Findings
1). Kaku et al, 2010	264 patients on a SU^a^ (glibenclamide, glicazide or glimeprimide)	Liraglutide 0.6 mg daily or 0.9 mg daily	Placebo with no active ingredients	1). Change of HbA1c at 24 wks	Japanese	−1.46% to −1.56% (liraglutide)vs. −0.40% (placebo)
				2). Proportion reaching HbA1c<7.0%		46.5% to 71.3% (liraglutide) vs. 14.8% (placebo)
2). Seino et al, 2012b	267 patients aged ≥20 years with suboptimal glycaemic control (HbA1c 7.4% to <10.4%)	Liraglutide 0.6 mg or 0.9 mg daily	Placebo with no active ingredients	Change of HbA1c at 24 wks	Japanese	Mean HbA1c was reduced by 1.00% to 1.27% points than placebo.
3). Kadowaki et al, 2011	179 patients who had suboptimal glycaemic control despite use of SU^a^ alone or in combination with a biguanide or thiazolidinedione	Exenatide 5 µg BID or 10 µg BID offered subcutaneously	Placebo with no active ingredients	Change of HbA1c at 24 wks	Japanese	−1.34% to −1.62% (exenatide) vs. −0.28% (p<0.001)
4). Inagaki et al, 2012	427 patients aged ≥20 years with insufficient glucose control	Exenatide QW 2 mg daily	Once daily insulin glarigine (starting dose 4U)	Change of HbA1c at 26 wks	Japanese	−1.11% (exenatide QW) vs. −0.68% (insulin Glargine), p<0.001 with the 95% C.I. upper limit <predefined non-inferiority margin (0.4%)
5). Seino et al, 2012a	311 patients aged 25–81 years with Hba1c between 7–10% and on basal insulin +/− SU^a^	Lixisenatide starting from 10 µg to 15 µg and 20 µg	Placebo with no active ingredients	Change of HbA1c at 24 wks	Japan, Republic of Korea, Taiwan, the Phillipines	−0.77% (lixisenatide) vs. 0.11% (placebo) (p<0.0001)
6). Inagaki et al, 2013	618 patients aged ≥20 years on SU^a^ or A-GI^b^	Linagliptin 5 mg daily	Metformin BD or TDS, up to 2,250 mg/day	1). Change of HbA1c at 52 wks	Japanese	1). −0.7% to −0.9% (linagliptin) vs. −0.8% to −1.0% (metformin), p = NS^c^
				2).Hypoglycaemic attack rates		2). 1.6% to 13.7% (linagliptin) vs. 3.2% to 15.9% (metformin); p = NS^c^
7). Zeng et al, 2013	192 patients on metformin and a SU^a^	Linagliptin 5 mg daily	Placebo with no active ingredients	1). Change of HbA1c at 24 wks	Chinese	1). −0.59% (linagliptin) vs. 0.08% (placebo), p<0.0001
				2). Change in FPG		2). −3.9 mg/dL (linagliptin) vs. 15.0% (placebo), p<0.001
				3). Adverse event rates		3). 38.9% (linagliptin) vs. 43.8% (placebo), p = NS^c^
8). Takihata et al, 2013	115 patients inadequately controlled with metformin and/or sulphonylurea	Sitagliptin 50 mg daily	Pioglitazone 15 mg daily	Change of HbA1c at 24 wks	Japanese	−0.86 (SD 0.63%) (sitagliptin) vs. −0.58 (SD 0.68%) (pioglitazone), p = 0.024
9). Seino et al, 2012	312 Patients on glimepiride (1–4 mg/day) with poor glycaemic control	Alogliptin 12.5 mg or 25 mg	Placebo with no active ingredients	1). Change of HbA1c at 12 wks	Japanese	1). −0.59% to −0.65% (alogliptin) vs. 0.35% (placebo)
				2). Adverse events		2). Comparable event rates with the majority being mild

a SU =  sulphonylurea.

b A-GI =  α-glucosidase inhibitors.

c NS =  Non-significant.

## Results

### Study characteristics

**Figure 1:**
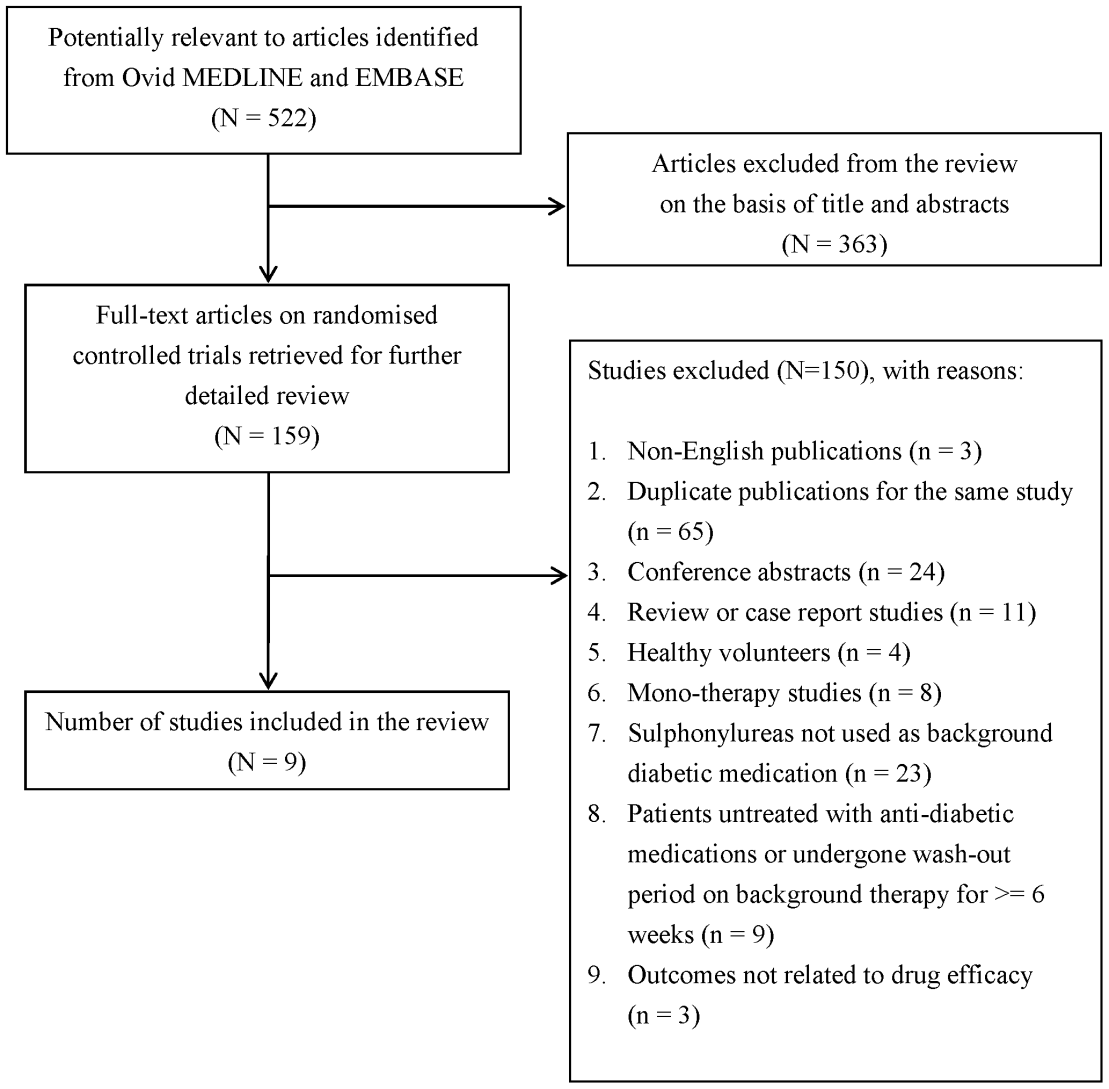
Flowchart of study selection.

A total of 522 articles were initially identified to be potentially relevant in MEDLINE and EMBASE. By reviewing the study titles and abstracts, 363 articles were excluded. Full-text articles were retrieved for the remaining 159 articles for formal inclusion and exclusion. Reasons for exclusion for the majority of trials included the duplicate publication of the same study, conference abstracts, and the absence of SUs treated prior to the medication of DPP-4 inhibitors and GLP-1 analogues. Nine studies fulfilled all the inclusion criteria and were finally included in this review. The details of the study selection and the reasons of exclusion were described in **Figure 1**.

### The effectiveness of GLP-1analogues as add-on therapies [48]–[52]

In a double-blind, parallel-group trial by Kaku et al. [48], 264 Japanese patients with type 2 diabetes were randomly given liraglutide, a GLP-1 analogue, with a dosage of 0.6 mg/day, 0.9 mg/day, or placebo combined with SU monotherapy. The mean changes in HbA1c from baseline to week 24 for liraglutide 0.6 mg, 0.9 mg and placebo were −1.46±0.95%, −1.56±0.84%, and −0.40±0.93%, demonstrating its dose-dependent improvement in glycaemic control in type 2 d'iabetic patients. Moreover, a higher proportion of subjects reached HbA1c <7.0% with liraglutide 0.6 mg (46.5%) or 0.9 mg (71.3%) than placebo (14.8%). In terms of safety, there were minor hypoglycaemic and gastrointestinal AEs, but no major hypoglycaemic events were reported.

Similar findings about GLP-1 analogue liraglutide were reported in one of the trials described in a recent study by Seino et al. [49]. In this trial, 267 Japanese type 2 diabetic patients were randomized to one of two daily doses of liraglutide (0.6 or 0.9 mg), or placebo while continuing SU treatment. Efficacy of the add-on liraglutide therapy to SU treatment was indicated by the reduction of the mean HbA1c of 1.00% and 1.27% point in the 0.6 mg and 0.9 mg liraglutide groups when compared to the placebo [49].

Another GLP-1 agonist, exenatide, was studied and analyzed for its treatment effect and safety in a study involving 179 Japanese patients with type 2 diabetes. The mean changes in HbA1c from baseline to week 24 were −1.34±0.11%, −1.62±0.11%, and −0.28±0.15% for exenatide 5 µg, 10 µg, and placebo, respectively (both p<0.001, exenatide vs. placebo) [50]. This suggested an apparent dose-response relationship in improving glycaemic control by exenatide. Common adverse effects of exenatide included hypoglycemia, nausea, and vomiting; however, these effects diminished over time when patients continued the treatment.

Another study by Inagaki et al. confirmed the treatment effect and safety of exenatide with 427 Japanese diabetic patients [51]. Patients were randomly assigned to add exenatide or insulin glargine to their current treatment. Patients treated with exenatide achieved a mean change of −1.11±0.06% in HbA1c from baseline to week 26, which was significantly greater than that in the insulin glargine group (between group difference: −0.43%, p<0.001). The proportion of subjects who reached HbA1c<7.0% with exenatide was significantly higher than those with insulin glargine (42.2% vs. 21.0%, p<0.001). Significantly less patients treated with exenatide reported symptoms of hypoglycaemia (9.3% vs. 19.8%, p = 0.002), and nocturnal hypoglycaemic episodes (0.9% vs. 10.4%, p<0.001) when compared with insulin glargine group.

A third type of GLP-1 agonist, lixisenatide, was examined in a randomized, double-blind, placebo-controlled trial by Seino et al. [52]. This study consisted of 311 patients with type 2 diabetes from four Asian countries, including Japan, the Republic of Korea, Taiwan, and the Philippines. These patients were on stable basal insulin therapy with (70%) or without (30%) SUs at screening. They were randomized into a lixisenatide or a placebo group for 24 weeks. The mean change in HbA1c from baseline to endpoint in the lixisenatide group was −0.77%, which was significantly different from that in the placebo group (between group difference: −0.88%, p<0.0001). In addition, significantly greater proportion of patients in this group reached HbA1c <7.0% (35.6% vs. 5.2%, p<0.0001). Though gastrointestinal and hypoglycaemic episodes were more common in the lixisenatide group when compared with the placebo, none of these events were severe. A few subjects withdrew due to nausea and vomiting.

### The effectiveness of DPP-4 inhibitors as add-on therapies [53]–[56]

A well-known DDP-4 inhibitor, linagliptin, has been supported to be as effective as metformin in providing add-on therapy for HbA1c reduction. In the 52-week, open-label, multicenter, parallel-group study conducted by Inagaki et al. [53], long-term efficacy of once-daily linagliptin 5 mg as add-on therapy to one oral antidiabetic drug [biguanide, glinide, glitazone, SUs or *α*-glucosidase inhibitors (A-GI)] as background therapy was evaluated in 618 Japanese patients with type 2 diabetes mellitus. At week 52, patients in the linagliptin group with SU background treatment achieved a significant reduction of 0.7% in mean HbA1c level, and 31.4% of these patients achieved the therapeutic target of HbA1c <7.0%. No significant difference in change in HbA1c from baseline was observed between linagliptin add-on to SUs and metformin add-on to SUs. Frequency of reported AEs was also similar between these two treatment groups among patients receiving SU background therapy [53].

The efficacy of linagliptin was further supported by a randomized, placebo-controlled, 24-week trial in China [54]. Once-daily linagliptin 5 mg was administered as add-on therapy to metformin and SUs in 192 Chinese patients with type 2 diabetes. The placebo-corrected mean change in HbA1c from baseline was −0.68% (p<0.0001) with linagliptin-based treatment at week 24. However, concerning the safety and tolerability, though overall AE rates were similar between the linagliptin and placebo groups (38.9% vs. 43.8%), drug-related AEs were higher in linagliptin group than in placebo group (12.5% vs. 2.1%) and the difference was due to hypoglycaemia (10.4% vs. 0.0%) [54].

The COMPASS study by Takihata et al. [55] also demonstrated sitagliptin, another DPP-4 inhibitor, was effective in improving glycaemic control when used as an add-on therapy. This was a multicenter, open-label, randomized controlled trial among 155 diabetic patients in Japan, comparing the effectiveness of sitagliptin with pioglitazone as add-on therapy to metformin and/or SUs. Among patients receiving sitagliptin, the mean changes in HbA1c level from baseline were −0.68±0.46%, −0.90±0.60%, and −0.86±0.63% at 8, 16 and 24 weeks respectively. There was a low incidence of AEs including hypoglycaemia in this treatment group throughout the study period [55].

Furthermore, Seino et al. [56] showed that add-on therapy of alogliptin to glimepiride in 312 Japanese diabetic patients achieved significant reduction in HbA1c. Patients receiving alogliptin once daily of 12.5 mg and 25 mg as add-on therapy to glimepiride had significant decrease in HbA1c (−0.59±0.058% and −0.65±0.059% respectively; p<0.0001) when compared with glimepiride monotherapy (0.35±0.059%, p<0.0001). Comparable number of AEs was reported among the three groups [56]. During the 40-week open-label extension study, the mean changes from baseline in HbA1c for the alogliptin 12.5 mg and 25 mg groups were significant at all time-points, with HbA1c values reduced by 0.42% and 0.58% respectively at the end of the study [56].

## Discussion

Nine studies from Asia-Pacific countries, involving a total of 2,685 type 2 diabetic patients, were included in this review. Duration of the exposure to the drug treatments varied with studies, ranging from 12 weeks to 52 weeks. Though heterogeneities in treatment protocol and drug dosage existed, results showed that add-on therapy of GLP-1 analogues (liraglutide, exenatide, and lixisenatide) and DPP-4 inhibitors (linagliptin, sitagliptin, and aloglitpin) were effective in improving glycaemic control among type 2 diabetic patients. This conclusion was supported by the reduction in HbA1c levels at the study endpoint, and the greater proportion of patients achieved HbA1c targets after the treatment. These results were consistent with previous Cochrane reviews on GLP-1 analogues and DPP-4 inhibitors [40], [57]. The Cochrane reviews showed favourable effects of GLP-1 analogues and DPP-4 inhibitors on reduction of HbA1c levels, though most of the included studies were from Western countries.

The current study focused on diabetic patients from Asia-Pacific region. This was of particular interest as the risk factors for diabetes differed between ethnic groups. Existing evidence suggested that patients from Asia had a high risk of type 2 diabetes when compared to the Western counterparts at the same BMI. They were more prone to abdominal obesity and low muscle mass with increased insulin resistance [58]. In addition, racial differences in response to drug treatment were identified in the literature. Review by Kim Y. G. [59] reported that when DPP-4 inhibitors were used in oral combination therapy, Asian-dominant studies showed a greater HbA1c lowering effect than non-Asian-dominant studies (between group difference: −0.18%, p = 0.006). All these data highlighted the importance of examining the efficacy of the two novel drugs as add-on therapies in diabetic patients from Asia-Pacific countries.

Despite current findings showed improvement in glycaemic control by this drug class, there was inconclusive information regarding its long term cardiovascular benefits. SAVOR [60], [61], which was a multicenter, randomized, double-blind, placebo-controlled, phase 4 trial conducted in 26 countries, reported a lower HbA1c level in patients receiving saxagliptin when compared to the placebo group. This study included a total of 16,496 diabetic patients from mainly Western countries (91.7%). Despite significant improvements in glycaemic control were observed, this study failed to show any cardioprotective benefit. Saxagliptin was found to be noninferior to placebo for the cardiovascular endpoints [61]. Similar results were reported in the recent EXAMINE trial. This trial recruited patients from different geographic regions, in which Asia/Pacific region accounted for around 20% of the total study population. No difference was observed in the primary end points of cardiovascular death, myocardial infarction, and stroke between the alogliptin and placebo groups. In particular, the rates of primary end point among subjects in Asia/Pacific region were 9.1% and 10.3% in alogliptin and placebo respectively (95%CI: 0.59–1.30) [62].

To summarize, the findings from these studies supported the efficacy and acceptable safety profiles of these two newer agents as add-on therapies to SUs and/or other medications among diabetic patients not having optimal glycaemic control. Nevertheless, some of the limitations of these studies should be mentioned. Firstly, almost all studies were conducted among Japanese patients and few representations from other ethnic groups were available in existing literature. This could be explained by the fact that Japanese type 2 diabetic patients are in general less obese with the ‘thrifty’ genotype, which causes more insulin deficiency and less insulin resistance than the Western population [63]. In fact, in a cross-sectional study of 17,000 Japanese type 2 diabetic patients, 72–78% on OHAs were using SUs [64]. Therefore, more research is needed in order to evaluate the efficacy and safety profile of incretin mimetics and DPP-4 inhibitors as add-on therapies for type 2 diabetes in other ethnic groups in the Asia-Pacific region as different ethnicities might have different pharmacological responses to these novel agents. For evaluation of their safety profiles, larger-scale studies are required as the AE rates may be so low - necessitating more subject enrolment to ensure the studies are adequately powered.

## Supporting Information

Checklist S1 **PRISMA Checklist.**
(DOCX)
